# Clinical and Economic Burden of Carbapenem-Resistant Infection or Colonization Caused by *Klebsiella pneumoniae*, *Pseudomonas aeruginosa*, *Acinetobacter baumannii*: A Multicenter Study in China

**DOI:** 10.3390/antibiotics9080514

**Published:** 2020-08-13

**Authors:** Xuemei Zhen, Cecilia Stålsby Lundborg, Xueshan Sun, Shuyan Gu, Hengjin Dong

**Affiliations:** 1 Centre for Health Management and Policy Research, School of Public Health, Cheeloo College of Medicine, Shandong University, (NHC Key Lab of Health Economics and Policy Research, Shandong University), Jinan 250012, China; zhenxuemei@sdu.edu.cn; 2 Center for Health Policy Studies, School of Public Health, Zhejiang University School of Medicine, Hangzhou 310058, China; sunxueshan@zju.edu.cn (X.S.); gushuyan@zju.edu.cn (S.G.); 3 Department of Global Public Health, Karolinska Institutet, 17177 Stockholm, Sweden; cecilia.stalsby.lundborg@ki.se; 4 Center for Health Policy and Management Studies, School of Government, Nanjing University, Nanjing 210023, China

**Keywords:** Carbapenem resistant *Klebsiella pneumoniae*, Carbapenem resistant *Pseudomonas aeruginosa*, Carbapenem resistant *Acinetobacter baumannii*, clinical burden, economic burden, China

## Abstract

**Background:** Carbapenem resistant *Klebsiella pneumoniae* (CRKP), *Pseudomonas aeruginosa* (CRPA), and *Acinetobacter baumannii* (CRAB) pose significant threats to public health. However, the clinical and economic impacts of CRKP, CRPA, and CRAB remain largely uninvestigated in China. This study aimed to examine the clinical and economic burden of CRKP, CRPA, and CRAB compared with carbapenem susceptible cases in China. **Method:** We conducted a retrospective and multicenter study among inpatients hospitalized at four tertiary hospitals between 2013 and 2015 who had *K. pneumoniae*, *P. aeruginosa*, and *A. baumannii* positive clinical samples. Propensity score matching (PSM) was used to balance the impact of potential confounding variables, including age, sex, insurance, number of diagnosis, comorbidities (disease diagnosis, and Charlson comorbidity index), admission to intensive care unit, and surgeries. The main indicators included economic costs, length of stay (LOS), and mortality rate. **Results:** We included 12,022 inpatients infected or colonized with *K. pneumoniae*, *P. aeruginosa*, and *A. baumannii* between 2013 and 2015, including 831 with CRKP and 4328 with carbapenem susceptible *K. pneumoniae* (CSKP), 1244 with CRPA and 2674 with carbapenem susceptible *P. aeruginosa* (CSPA), 1665 with CRAB and 1280 with carbapenem susceptible *A. baumannii* (CSAB). After PSM, 822 pairs, 1155 pairs, and 682 pairs, respectively were generated. Compared with carbapenem-susceptible cases, those with CRKP, CRPA, and CRAB were associated with statistically significantly increased total hospital cost ($14,252, *p* < 0.0001; $4605, *p* < 0.0001; $7277, *p* < 0.0001) and excess LOS (13.2 days, *p* < 0.0001; 5.4 days, *p* = 0.0003; 15.8 days, *p* = 0.0004). In addition, there were statistically significantly differences in hospital mortality rate between CRKP and CSKP, and CRAB and CSAB group (2.94%, *p* = 0.024; 4.03%, *p* = 0.03); however, the difference between CRPA and CSPA group was marginal significant (2.03%, *p* = 0.052). **Conclusion:** It highlights the clinical and economic impact of CRKP, CRPA, and CRAB to justify more resources for implementing antibiotic stewardship practices to improve clinical outcomes and to reduce economic costs.

## 1. Introduction

Carbapenem resistance poses a significant threat to public health globally. It occurs mainly among gram-negative bacteria such as *Klebsiella pneumoniae*, *Pseudomonas aeruginosa*, and *Acinetobacter baumannii*, which are the most concerning bacteria with a propensity toward multi-drug resistance and cause the life-threatening healthcare associated infections amongst critically ill and immunocompromised individuals [1]. Carbapenem resistant *K. pneumoniae* (CRKP), *P. aeruginosa* (CRPA), and *A. baumannii* (CRAB) were recognized as critical-priority bacteria, by the World Health Organization (WHO), which was mainly based on the associated increasing clinical and economic burden, unavailability of effective therapy, and antibiotic resistant characteristics [2].

In the European countries, the population-weighted mean proportions of CRKP, CRPA, and CRAB were 7.5%, 17.2%, and 31.9% in 2018, respectively [3]. In China, there were marked increases in the proportion of CRKP from 3.0% in 2005 to 26.3% in 2018, and in the prevalence of CRAB from 39.0% in 2005 to 73.9% in 2018. The proportion of CRPA showed a downward trend during between 2005 and 2014, but deteriorated from 2015 and reached 30.7% in 2018 [4,5,6,7,8].

Recently published studies mainly focus on gram-positive bacteria [9], while only few studies estimated the burden of CRKP, CRPA, and CRAB in terms of mortality, length of stay (LOS), and cost [10,11,12,13,14]. It was reported that patients with CRKP, CRPA, and CRAB infections were associated with higher mortality, longer hospital stay, and higher hospital costs compared with carbapenem susceptible cases [9,15,16,17]. In addition, hospital costs incurred by colonized patients with CRKP were also high [18]. However, it was also found that there were no significant differences in hospital mortality between CRPA and carbapenem susceptible *P. aeruginosa* (CSPA) cases [10,15], in total hospital cost between CRAB and carbapenem susceptible *A. baumannii* (CSAB) cases [14].

In China, there were some studies exploring the mortality and LOS associated with CRKP [19,20,21,22,23], CRPA [24,25,26], or CRAB [27,28,29,30], and exploring the economic burden attributed by CRKP [19], CRPA [24], or CRAB [27,31]. Although hospitalized patients with carbapenem resistance appeared to have increased hospitality mortality, hospital stay, and hospital costs, no significant differences in hospital mortality and hospital stay between CRKP and carbapenem susceptible *K. pneumoniae* (CSKP) cases were showed as well [19,21]. In addition, majority of studies were conducted in a single hospital setting, and using univariate analyses regardless of confounding risk factors [32]. Therefore, there are limited published data and the clinical and economic impacts of CRKP, CRPA, and CRAB remain largely uninvestigated in China. In this study, we aimed to examine the clinical and economic burden of carbapenem resistance in *K. pneumoniae*, *P. aeruginosa*, and *A. baumannii* compared with carbapenem susceptible cases in China.

## 2. Material and Methods

### 2.1. Study Site

This study was conducted in four tertiary hospitals in China. Hospital site 1 and site 2 are general provincial hospitals in Zhejiang province and Shandong province, respectively. Hospital site 3 and site 4 are general county hospital and combined traditional Chinese and Western medicine provincial hospital in Zhejiang province, respectively. Hospital site 1–4 are with 3200, 3500, 1727, 2100 of hospital beds, and with 170,000, 160,000, 80,000, 50,000 of inpatients per year, respectively.

### 2.2. Study Design and Patients

It was a retrospective and multicenter study. Patients were identified from the records of the clinical microbiology laboratory. 100% of inpatients in hospital site 2–4, and 60% of inpatients in hospital site 1 between 2013–2015 who had clinical samples positive for CRKP, CRPA, CRAB infection or colonization were included. The control group comprised of patients with CSKP, carbapenem susceptible *P. aeruginosa* (CSPA), and CSAB-positive clinical samples with the same percentages who were hospitalized during the same study period. Only 60% of inpatients were randomly selected from hospital site 1 due to the large inpatient population [31]. The interpretation of carbapenem susceptibility was based on the Clinical and Laboratory Standards Institute (CLSI) definitions and reported as resistant (R), susceptible (S), and intermediate (I). *K. pneumoniae*, *P. aeruginosa*, and *A. baumannii* isolates, were considered resistant to carbapenems if they were resistant or intermediate to any carbapenem (imipenem, meropenem, panipenem or ertapenem) [31], and the control group was defined as patients infected or colonized by *K. pneumoniae*, *P. aeruginosa*, and *A. baumannii* susceptible to all carbapenems. To avoid duplication, cases with the first episode detected in any clinical specimen (e.g., blood, stool, cervical, urethral sources) were included. If multiple samples were taken from a patient during the study period, only the first sample was included in this study.

### 2.3. Data Collection

Data were obtained from patients’ medical records. Patients’ characteristics included demographics (age, sex, and insurance), comorbidities (disease diagnosis, and Charlson comorbidity index (CCI), hospital events (admitting service, surgical services, and dates of hospital and intensive care unit (ICU) admission/discharge), and clinical outcomes (discharged alive or death during hospitalization). We also noted any related laboratory results when isolation of *K. pneumoniae*, *P. aeruginosa*, and *A. baumannii* was recorded in the inspection system, and recorded the antibiotic susceptibility test results obtained from the microbiology laboratory. In addition, economic costs associated with these patients were obtained from the financial system. The economic costs included total hospital cost, medication cost (antibiotic cost), diagnostic cost, treatment cost, material cost, and other costs, including out-of pocket payment and payment covered by health insurers. Values of the economic costs were converted from Chinese Yuan to United States dollars in 2015 [33,34].

### 2.4. Propensity Score Matching

To eliminate the impact of potential confounding variables, propensity score matching (PSM) with 1:1 nearest-neighbor matching using STATA was conducted. For logistic regression modeling with carbapenem resistant and carbapenem susceptible as dependent variables, independent variables included age, sex, insurance, number of diagnosis, CCI, admission to ICU, surgery, and comorbidities, but not clinical and economic outcomes (economic costs, LOS, and in-hospital mortality). The generated pairs, who were matched for all included variables, were subjected to further analyses of economic costs, LOS, and in-hospital mortality.

### 2.5. Indicators and Statistical Analyses

In this study, the main indicators included economic costs, LOS, and in-hospital mortality. We performed 1000 iterations of Monte Carlo simulations to calculate the 95% uncertainty interval for each indicator with normal distribution using SAS [35]. We compared the main indicators between CRKP and CSKP group, between CRPA and CSPA group, between CRAB and CSAB group using *t* test and χ^2^ test for quantitative and qualitative variables, respectively. All *p*-values were two-tailed, <0.05 were deemed statistically significant, and ≥0.05 and <0.10 were considered marginally significant.

### 2.6. Ethical Approval and Informed Consent

The study was approved by the institutional review board of Zhejiang University School of Public Health, who waived the need for informed consent. All inpatients data were anonymized prior to analysis.

## 3. Results

We included 12,022 inpatients infected or colonized with *K. pneumoniae*, *P. aeruginosa*, and *A. baumannii* between 2013 and 2015, including 5159 *K. pneumoniae*, 3918 *P. aeruginosa*, and 2945 *A. baumannii*. Of these, a total of 831 with CRKP and 4328 with CSKP, 1244 with CRPA and 2674 with CSPA, 1665 with CRAB and 1280 with CSAB were included. Significant differences were found in insurance, admission to ICU, surgery between CRKP and CSKP group, in age, sex, number of diagnosis, CCI, admission to ICU between CRPA and CSPA group, in age, sex, insurance, number of diagnosis, CCI, admission to ICU, surgery between CRAB and CSAB group. Some comorbidities between the three groups were significantly different as well. After PSM, there were no differences in patients’ characteristics between the three groups, and generated 822 pairs, 1155 pairs, and 682 pairs, respectively (Table 1, Table 2 and Table 3).

Inpatients with carbapenem resistance were significantly associated with higher economic costs than carbapenem susceptible cases. For patients with *K. pneumoniae*, the mean differences (95% UI) in total hospital cost, antibiotic cost, medication cost including antibiotics, diagnostic cost, treatment cost and material cost were $14,251 ($13,852–$14,653), $3296 ($3202–$3390), $9132 ($8910–$9354), $1517 ($1465–$1570), $2574 ($2462–$2686), and $899 ($826–$971), respectively (Table 4). For patients with *P. aeruginosa*, the mean differences (95% UI) in economic costs were $4,605 ($4249–$4960), $1078 ($1007–$1149), $3053 ($2867–$3238), $370 ($323–416), $1174 ($1043–$1305), and $76 ($19–$132), respectively (Table 5). For patients with *A. baumannii*, the mean differences (95% UI) in economic costs were $7277 ($6897–$7657), $1537 ($1468–$1605), $3902 ($3731–$4074), $628 ($585–$670), $2156 ($1967–$2344), $421 ($351–$491), respectively (Table 6).

Compared with inpatients with CSKP, CSPA, and CSAB, those with CRKP, CRPA, and CRAB were significantly associated with longer LOS, with mean differences (95% UI) of 13.2 days (12.7–13.7days), 5.4 days (4.4–6.5 days), and 15.8 days (13.9–17.7 days), respectively (Table 7).

There were statistical differences in in-hospital mortality rate between CRKP and CSKP group (9.59% (9.33–9.85%) vs. 6.65% (6.43–6.87%)), and CRAB and CSAB group (8.28% (8.04–8.53%) vs. 4.25% (4.07–4.43%)). The difference in in-hospital mortality rate between CRPA and CSPA group was marginal significant, with the difference rate of 2.03% (1.75–2.32%) (*p* = 0.052) (Table 8).

## 4. Discussion

To the best of our knowledge, this is the first large and multicenter study quantifying the clinical and economic impact of CRKP, CRPA, and CRAB in mainland China using the PSM method. We found that after PSM, compared with carbapenem susceptible cases, those with CRKP, CRPA, and CRAB were associated with significantly increased economic costs, excess LOS, and attributable in-hospital mortality rate. A marginal difference in hospital mortality rate existed between CRPA and CSPA group.

It was clearly demonstrated that economic costs for infection or colonization caused by carbapenem resistance were higher than in case of carbapenem susceptible bacteria, suggesting that carbapenem resistance indeed incurs excessive economic costs on patients infected or colonized with *K. pneumoniae*, *P. aeruginosa*, and *A. baumannii*. It also shows that the impact of carbapenem resistance on economic costs might depend on the type of gram-negative bacteria, with resistance in *K. pneumoniae* having a larger impact, followed by *A. baumannii* and *P. aeruginosa*. This finding is consistent with several previous studies, in which carbapenem resistance was associated with higher economic costs for infection or colonization caused by gram-negative bacteria, including *K. pneumonia* [19], *P. aeruginosa* [10,12,24], and *A. baumannii* [11,13,27,31]. However, one study did not find a statistically significant association in total hospital cost among infants with ventilator associated pneumonia in the ICU between CRAB and CSAB group [14], which might be because that critical illness can attenuate the effect of carbapenem resistance [36]. Effective control of carbapenem resistance would result in cost savings and could be useful for assessing the cost-effectiveness of interventions to reduce the development and spread of carbapenem resistance in hospital settings [19].

We indeed found that carbapenem resistance was associated with longer LOS, which is similar with other investigations that patients with resistant bacteria requires increases in the number of hospitalization days [19,24,37]. Prolonged LOS associated with carbapenem resistance might be independent of the type of gram-negative bacteria, with resistance in *A. baumannii* having a larger influence, followed by *K. pneumoniae*, and *P. aeruginosa*. Hospital stay is the major contributor to the additional economic costs in carbapenem resistant infection or colonization, as patients with longer hospital stay were associated with more antibiotic therapy and more surgeries [19,24,37]; therefore, LOS was not included in PSM analyses [38].

The poorest clinical outcomes were observed for CRAB, followed by CRKP and CRPA. In-hospital mortality rate was significantly higher for patients with CRKP and CRAB than for carbapenem susceptible cases, which is similar with other studies [16,39,40]. A 2.03% increase in mortality rate for the CRPA patients is clinically meaningful, and the non-significant *p*-value (*p* = 0.052) is too low to confidently rule out an effect of CRPA on mortality rate, which is different with other findings [16,39,40], however, several studies reported non-significant association between mortality and CRPA as well [10,15]. Spending on medical services was associated with a reduction in the mortality rate, therefore, it is critical to consider the clinical and economic burden posed by CRKP, CRPA, and CRAB when shirting resource allocation [41].

The proportions of CRKP, CRPA, and CRAB in the four sampled hospitals were 10.29%, 31.75%, and 56.54%, respectively, which were approximate the national levels reported in China Antimicrobial Resistance Surveillance System (CARSS) (8.03%, 22.61%, and 58.05%) [42]. In addition, the mean total hospital cost, length of hospital stay, and hospital mortality were $3042 and $2470, 10.1 days and 9.4 days, 0.3% and 0.4% in Zhejiang and Shandong province in 2015, respectively, which were similar to the national levels ($2378, 9.6 days, and 0.4%). Therefore, we assumed that the antibiotic resistant level, and clinical and economic burden due to CRKP, CRPA, and CRAB from four sampled hospitals in China were approximate results representing the national level.

Our study has some limitations. First, due to the retrospective nature of our study, it is difficult to distinguish infection or colonization, which may underestimate the clinical and economic outcome of carbapenem resistant infection. However, colonization is an important reservoir for bacteria causing infection, therefore, it is important to identify the burden of patients with CRKP, CRPA and CRAB, either infected or colonized, in order to contain the spread of these bacteria. In this study, we explored the clinical and economic outcomes for CRKP, CRPA and CRAB isolation and not specifically infection. Studies on clinical infection or colonization separate should be considered in the future as well. Secondly, this is a retrospective study, inherently resulting in bias and confounding, thus, PSM was conducted to balance potential confounding factors in order to minimize the risk of bias. However, PSM is not without its own limitations, which ignore unmeasured confounders that could potentially impact outcomes. Moreover, although it is a multicenter study that was conducted in four tertiary hospitals, the antibiotic resistant levels and the main indicators approximate the national levels, it is necessary to expand this study to different types of hospitals in different areas in the future. Finally, as we had data between 2013 and 2015 only, Monte Carlo simulations with 1000 iterations were conducted and mean values during the study period were reported. Although study period did not influence the conclusions, future studies with update data are warranted.

## 5. Conclusions

This study underscores the substantial clinical and economic burden associated with CRKP, CRPA, and CRAB in hospitalized patients. The input of more resources to control carbapenem resistance in *K. pneumoniae*, *P. aeruginosa*, and *A. baumannii* can be justified both for improving clinical outcomes, and for reducing economic costs, thus maximized benefit with available resources. In addition, urgent need for implementing antibiotic stewardship practices across the continuum of hospital settings will hopefully help to curb the emergency and spread of carbapenem resistance and *K. pneumoniae*, *P. aeruginosa*, and *A. baumannii*.

## Figures and Tables

**Table 1:** Characteristics of the patients with CRKP and CSKP before and after PSM.

Baseline Characteristics	Before PSM			After PSM		
	CSKP	CRKP	*p*-Value	CSKP	CRKP	*p*-Value
Number of inpatient, n	4328	831		822	822	
Age in years, median (range)	72 (0–100)	73 (0–98)	0.323	70 (0–100)	73 (0–98)	0.602
Sex male, n (%)	2963 (68.46)	554 (66.67)	0.309	554 (67.40)	547 (66.55)	0.714
Insurance, n (%)	3568 (82.44)	658 (79.18)	0.025	644 (78.35)	650 (79.08)	0.718
Number of diagnosis, median (range)	6 (1–30)	7 (1–23)	0.129	6 (1–30)	6.5 (1–23)	0.481
Charlson comorbidity index, median (range)	5 (1–34)	5 (1–18)	0.074	5 (1–24)	5 (1–18)	0.710
Admission to ICU, n (%)	503 (11.62)	321 (38.63)	<0.0001	310 (37.71)	312 (37.96)	0.919
Surgery, n (%)	1106 (25.55)	277 (33.33)	<0.0001	300 (36.50)	276 (33.58)	0.215
Myocardial infarction, n (%)	124 (2.87)	19 (2.29)	0.352	22 (2.68)	19 (2.31)	0.635
Congestive heart failure, n (%)	764 (17.65)	121 (14.56)	0.03	125 (15.21)	119 (14.48)	0.677
Peripheral vascular disease, n (%)	54 (1.25)	11 (1.32)	0.857	12 (1.46)	11 (1.34)	0.834
Cerebrovascular diseases, n (%)	2178 (50.32)	484 (58.24)	<0.0001	462 (56.20)	476 (57.91)	0.485
Dementia, n (%)	130 (3.00)	24 (2.89)	0.858	23 (2.80)	24 (2.92)	0.882
Chronic pulmonary disease, n (%)	1084 (25.05)	154 (18.53)	<0.0001	139 (16.91)	154 (18.73)	0.334
Connective tissue disease, n (%)	83 (1.92)	9 (1.08)	0.096	11 (1.34)	9 (1.09)	0.653
Mild liver disease, n (%)	163 (3.77)	24 (2.89)	0.215	25 (3.04)	24 (2.92)	0.885
Peptic ulcer disease, n (%)	29 (2.98)	31 (3.73)	0.253	40 (4.87)	31 (3.77)	0.275
Diabetes mellitus, n (%)	1206 (27.87)	194 (23.35)	0.007	175 (21.29)	193 (23.48)	0.287
Diabetes mellitus with chronic complications, n (%)	163 (3.77)	15 (1.81)	0.005	13 (1.58)	15 (1.82)	0.703
Moderate to severe chronic kidney disease, n (%)	322 (7.44)	102 (12.27)	<0.0001	116 (14.11)	97 (11.80)	0.163
Hemiplegia, n (%)	38 (0.88)	8 (0.96)	0.812	6 (0.73)	8 (0.97)	0.591
Solid tumor without metastases, n (%)	298 (6.89)	52 (6.26)	0.51	59 (7.18)	50 (6.08)	0.372
Leukemia, n (%)	67 (1.55)	24 (2.89)	0.007	27 (3.28)	24 (2.92)	0.670
Malignant lymphoma, n (%)	46 (1.06)	15 (1.81)	0.07	19 (2.31)	15 (1.82)	0.488
Severe liver disease, n (%)	57 (1.32)	13 (1.56)	0.572	11 (1.34)	13 (1.58)	0.681
Metastatic tumor, n (%)	249 (5.75)	16 (1.93)	<0.0001	15 (1.82)	16 (1.95)	0.856

CRKP: carbapenem resistant Klebsiella pneumoniae, CSKP: carbapenem susceptible K. pneumoniae, PSM: propensity score matching, ICU: intensive care unit.

**Table 2:** Characteristics of the patients with CRPA and CSPA before and after PSM.

Baseline Characteristics	Before PSM			After PSM		
	CSPA	CRPA	*p*-Value	CSPA	CRPA	*p*-Value
Number of inpatient, n	2674	1244		1155	1155	
Age in years, median (range)	73 (0–100)	79 (0–98)	<0.0001	77 (0–100)	79 (0–98)	0.994
Sex male, n (%)	1775 (66.38)	877 (70.50)	0.01	803 (69.52)	812 (70.30)	0.683
Insurance, n (%)	2322 (86.84)	1084 (87.14)	0.794	1007 (87.19)	1007 (87.19)	1.000
Number of diagnosis, median (range)	6 (1–36)	7 (1–21)	0.002	7 (1–36)	7 (1–21)	0.444
Charlson comorbidity index, median (range)	5 (1–34)	5 (1–22)	0.007	5 (1–34)	5 (1–22)	0.468
Admission to ICU, n (%)	282 (10.55)	283 (22.75)	<0.0001	252 (21.82)	221 (19.13)	0.110
Surgery, n (%)	554 (20.72)	275 (22.11)	0.322	272 (23.55)	245 (21.21)	0.178
Myocardial infarction, n (%)	65 (2.43)	46 (3.70)	0.026	40 (3.46)	40 (3.46)	1.000
Congestive heart failure, n (%)	504 (18.85)	196 (15.76)	0.019	192 (16.62)	178 (15.41)	0.427
Peripheral vascular disease, n (%)	24 (0.90)	20 (1.61)	0.05	19 (1.65)	17 (1.47)	0.737
Cerebrovascular diseases, n (%)	1360 (50.86)	813 (65.35)	<0.0001	732 (63.38)	735 (63.64)	0.897
Dementia, n (%)	169 (6.32)	86 (6.91)	0.484	82 (7.10)	85 (7.36)	0.810
Chronic pulmonary disease, n (%)	1113 (41.62)	292 (23.47)	<0.0001	268 (23.20)	289 (25.02)	0.307
Connective tissue disease, n (%)	55 (2.06)	16 (1.29)	0.092	17 (1.47)	16 (1.39)	0.861
Mild liver disease, n (%)	40 (1.50)	23 (1.85)	0.414	21 (1.82)	22 (1.90)	0.878
Peptic ulcer disease, n (%)	57 (2.13)	37 (2.97)	0.109	37 (3.20)	33 (2.86)	0.627
Diabetes mellitus, n (%)	614 (22.96)	310 (24.92)	0.179	309 (26.75)	291 (25.19)	0.393
Diabetes mellitus with chronic complications, n (%)	52 (1.94)	23 (1.85)	0.839	21 (1.82)	22 (1.90)	0.878
Moderate to severe chronic kidney disease, n (%)	191 (7.14)	105 (8.44)	0.153	93 (8.05)	94 (8.14)	0.939
Hemiplegia, n (%)	37 (1.38)	21 (1.69)	0.463	20 (1.73)	20 (1.73)	1.000
Solid tumor without metastases, n (%)	117 (4.38)	59 (4.74)	0.605	56 (4.85)	50 (4.33)	0.551
Leukemia, n (%)	21 (0.79)	15 (1.21)	0.199	13 (1.13)	11 (0.95)	0.682
Malignant lymphoma, n (%)	17 (0.64)	8 (0.64)	0.979	10 (0.87)	8 (0.69)	0.636
Severe liver disease, n (%)	23 (0.86)	9 (0.72)	0.658	11 (0.95)	8 (0.69)	0.490
Metastatic tumor, n (%)	70 (2.62)	24 (1.93)	0.19	24 (2.08)	23 (1.99)	0.883

CRKP: carbapenem resistant *Pseudomonas aeruginosa*, CSKP: carbapenem susceptible *P. aeruginosa*, PSM: propensity score matching, ICU: intensive care unit.

**Table 3:** Characteristics of the patients with CRAB and CSAB before and after PSM.

Baseline Characteristics	Before PSM			After PSM		
	CSAB	CRAB	*p*-Value	CSAB	CRAB	*p*-Value
Number of inpatient, n	1280	1665		682	682	
Age in years, median (range)	73 (0–100)	68 (0–102)	<0.0001	70.5 (0–100)	71 (0–102)	0.845
Sex male, n (%)	917 (71.64)	1127 (67.69)	0.021	480 (70.38)	480 (70.38)	1.000
Insurance, n (%)	1030 (80.47)	1167 (70.09)	<0.0001	500 (73.31)	510 (74.78)	0.537
Number of diagnosis, median (range)	6 (1–23)	6 (1–24)	0.02	6 (1–23)	6 (1–24)	0.243
Charlson comorbidity index, median (range)	5 (1–28)	4 (1–18)	<0.0001	5 (1–16)	5 (1–17)	0.352
Admission to ICU, n (%)	160 (12.50)	825 (49.55)	<0.0001	160 (23.46)	170 (24.93)	0.527
Surgery, n (%)	380 (29.69)	753 (45.23)	<0.0001	250 (36.66)	246 (36.07)	0.822
Myocardial infarction, n (%)	34 (2.66)	47 (2.82)	0.784	24 (3.52)	22 (3.23)	0.764
Congestive heart failure, n (%)	250 (19.53)	247 (14.83)	0.001	115 (16.86)	123 (18.04)	0.568
Peripheral vascular disease, n (%)	21 (1.64)	36 (2.16)	0.309	20 (2.93)	18 (2.64)	0.742
Cerebrovascular diseases, n (%)	676 (52.81)	1002 (60.18)	<0.0001	389 (57.04)	380 (55.72)	0.623
Dementia, n (%)	42 (3.28)	41 (2.46)	0.183	33 (4.84)	21 (3.08)	0.096
Chronic pulmonary disease, n (%)	342 (26.72)	306 (18.38)	<0.0001	138 (20.23)	163 (23.90)	0.103
Connective tissue disease, n (%)	21 (1.64)	22 (1.32)	0.474	10 (1.47)	6 (0.88)	0.314
Mild liver disease, n (%)	42 (3.28)	56 (3.36)	0.902	26 (3.81)	22 (3.23)	0.557
Peptic ulcer disease, n (%)	34 (2.66)	51 (3.06)	0.513	21 (3.08)	20 (2.93)	0.874
Diabetes mellitus, n (%)	295 (23.05)	354 (21.26)	0.247	160 (23.46)	152 (22.29)	0.606
Diabetes mellitus with chronic complications, n (%)	39 (3.05)	19 (1.14)	<0.0001	10 (1.47)	14 (2.05)	0.410
Moderate to severe chronic kidney disease, n (%)	100 (7.81)	160 (9.61)	0.088	61 (8.94)	54 (7.92)	0.495
Hemiplegia, n (%)	15 (1.17)	31 (1.86)	0.134	12 (1.76)	11 (1.61)	0.833
Solid tumor without metastases, n (%)	121 (9.45)	72 (4.32)	<0.0001	32 (4.69)	45 (6.60)	0.127
Leukemia, n (%)	18 (1.41)	4 (0.24)	<0.0001	2 (0.29)	3 (0.44)	1.000
Malignant lymphoma, n (%)	13 (1.02)	7 (0.42)	0.051	1 (0.15)	3 (0.44)	0.624
Severe liver disease, n (%)	15 (1.17)	25 (1.50)	0.444	11 (1.61)	6 (0.88)	0.222
Metastatic tumor, n (%)	94 (7.34)	26 (1.56)	<0.0001	12 (1.76)	21 (3.08)	0.113

CRKP: carbapenem resistant *Acinetobacter baumannii*, CSKP: carbapenem susceptible *A. baumannii*, PSM: propensity score matching, ICU: intensive care unit.

**Table 4:** Economic costs of patients with CRKP and CSKP after PSM for potential confounding variables.

Hospital Cost ($)	CSKP			CRKP			Difference			*p*-Value
	Mean	95% UI		Mean	95% UI		Mean	95% UI		
Total hospital cost	21,229	21,005	21,453	35,480	35,149	35,811	14,252	13,852	14,653	<0.0001
Antibiotic cost	2878	2824	2932	6175	6097	6252	3296	3202	3390	<0.0001
Medication cost	10,081	9960	10,202	19,213	19,027	19,399	9132	8910	9354	<0.0001
Diagnostic cost	2868	2836	2900	4385	4343	4426	1517	1465	1570	<0.0001
Treatment cost	5112	5047	5176	7686	7595	7776	2574	2462	2686	<0.0001
Material cost	3096	3051	3140	3993	3937	4050	899	826	971	<0.0001
Other cost	70	68	73	62	62	64	−8	−11	−5	0.0028

CRKP: carbapenem resistant *Klebsiella pneumoniae*, CSKP: carbapenem susceptible *K. pneumoniae*, PSM: propensity score matching, UI: uncertainty interval.

**Table 5:** Economic costs of patients with CRPA and CSPA after PSM for potential confounding variables.

Hospital Cost ($)	CSPA			CRPA			Difference			*p*-Value
	Mean	95% UI		Mean	95% UI		Mean	95% UI		
Total hospital cost	20,908	20,670	21,146	25,508	25,244	25,771	4605	4249	4960	<0.0001
Antibiotic cost	2426	2380	2472	3504	3450	3558	1078	1007	1149	<0.0001
Medication cost	10,082	9959	10,203	13,135	12,996	13,273	3053	2867	3238	<0.0001
Diagnostic cost	2759	2728	2790	3129	3094	3163	370	323	416	0.0001
Treatment cost	5512	5434	5591	6683	6578	6789	1174	1043	1305	<0.0001
Material cost	2397	2354	2440	2472	2435	2508	76	19	132	<0.0001
Other cost	62	60	63	84	81	87	23	19	26	0.3252

CRKP: carbapenem resistant *Pseudomonas aeruginosa*, CSKP: carbapenem susceptible *P. aeruginosa*, PSM: propensity score matching, UI: uncertainty interval.

**Table 6:** Economic costs of patients with CRAB and CSAB after PSM for potential confounding variables.

Hospital Cost ($)	CSAB			CRAB			Difference			*p*-Value
	Mean	95% UI		Mean	95% UI		Mean	95% UI		
Total hospital cost	20,349	20,103	20,595	27,630	27,342	27,919	7277	6897	7657	<0.0001
Antibiotic cost	2380	2331	2428	3917	3868	3965	1537	1468	1605	<0.0001
Medication cost	9160	9036	9283	13,065	12,948	13,183	3902	3731	4074	<0.0001
Diagnostic cost	2840	2809	2870	3468	3439	3497	628	585	670	<0.0001
Treatment cost	5155	5073	5237	7313	7143	7482	2156	1967	2344	<0.0001
Material cost	3102	3050	3153	3521	3473	3569	421	351	491	<0.0001
Other cost	91	89	93	93	90	96	2	−2	6	0.0001

CRAB: carbapenem resistant *Acinetobacter baumannii*, CSAB: carbapenem susceptible *A. baumannii*, PSM: propensity score matching, UI: uncertainty interval.

**Table 7:** Length of stay among patients with CRKP and CSKP, among patients with CRPA and CSPA, and among patients with CRAB and CSAB after PSM for potential confounding variables.

Length of Stay (Days)	Carbapenem Susceptible-			Carbapenem Resistant-			Difference			*p*-Value
	Mean	95% UI		Mean	95% UI		Mean	95% UI		
CRKP vs. CSKP	32.9	32.6	33.2	46.1	45.7	46.5	13.2	12.7	13.7	<0.0001
CRPA vs. CSPA	41.1	40.6	41.6	46.5	45.6	47.4	5.4	4.4	6.5	0.0003
CRAB vs. CSAB	33.7	33.4	34.1	49.6	47.7	51.4	15.8	13.9	17.7	0.0004

CRKP: carbapenem resistant *Klebsiella pneumoniae*, CSKP: carbapenem susceptible *K. pneumoniae*, CRKP: carbapenem resistant *Pseudomonas aeruginosa*, CSKP: carbapenem susceptible *P. aeruginosa*, CRAB: carbapenem resistant *Acinetobacter baumannii*, CSAB: carbapenem susceptible *A. baumannii*, PSM: propensity score matching, UI: uncertainty interval.

**Table 8:** In hospital mortality among patients with CRKP and CSKP, among patients with CRPA and CSPA, and among patients with CRAB and CSAB after PSM for potential confounding variables.

In Hospital Mortality Rate (%)	Carbapenem Susceptible-			Carbapenem Resistant-			Difference			*p*-Value
	Rate	95% UI		Rate	95% UI		Rate	95% UI		
CRKP vs. CSKP	6.65	6.43	6.87	9.59	9.33	9.85	2.94	2.6	3.28	0.024
CRPA vs. CSPA	4.73	4.54	4.91	6.77	6.55	6.99	2.03	1.75	2.32	0.052
CRAB vs. CSAB	4.25	4.07	4.43	8.28	8.04	8.53	4.03	3.73	4.33	0.003

CRKP: carbapenem resistant *Klebsiella pneumoniae*, CSKP: carbapenem susceptible *K. pneumoniae*, CRKP: carbapenem resistant *Pseudomonas aeruginosa*, CSKP: carbapenem susceptible *P. aeruginosa*, CRAB: carbapenem resistant *Acinetobacter baumannii*, CSAB: carbapenem susceptible *A. baumannii*, PSM: propensity score matching, UI: uncertainty interval.
